# Case report: Left ventricular perforation caused by right ventricular pacemaker lead

**DOI:** 10.3389/fcvm.2022.1089694

**Published:** 2023-01-12

**Authors:** Xiang Huang, Heng Zhou, Xiao-Mei Li, Xiao-Lan Li

**Affiliations:** Department of Cardiology, Xiangyang No.1 People’s Hospital, Hubei University of Medicine, Xiangyang, Hubei, China

**Keywords:** pacemaker, lead migration, left ventricular perforation, transvenous lead extraction, right ventricular pacemaker lead

## Abstract

**Background:**

Perforation of the interventricular septum and left ventricular (LV) free wall by a right ventricular (RV) lead is an extremely rare and potentially life-threatening complication. In this case report, we discussed the diagnosis and management of a very unusual complication of pacemaker (PM) implantation, i.e., LV perforation brought on by an RV pacing lead.

**Case summary:**

A 92-year-old man was admitted to Xiangyang No.1 People’s Hospital due to a complete atrioventricular block. We performed a dual-chamber PM implantation; however, on the second postoperative day (POD), pacemaker failure occurred. Thoracic computed tomography (CT) scan showed that RV lead had pierced the interventricular septum and LV free wall. A transvenous lead extraction of the penetrating lead was performed uneventfully, and RV lead was refixed at the lower RV septum on the 5th POD.

**Discussion:**

Identification of high-risk patients is mandatory to prevent this serious complication, and transvenous lead extraction with cardiac surgery backup may be an option.

## 1. Introduction

Iatrogenic ventricle perforation by pacing/defibrillation leads is a rare but potentially life-threatening complication which occurs in only 0.3–0.8% of pacemaker (PM) or implantable cardioverter defibrillator (ICD) implantation procedures (1). Although the right ventricular (RV) apex is the site of lead perforation that occurs most frequently, few cases have been reported of lead penetration through the interventricular septum and the left ventricular (LV) free wall to the left hemithorax (2). Appropriate management of lead migration in the left hemithorax is still uncertain. Among these reported cases, most patients underwent surgical extraction of the penetrating lead (3).

## 2. Case presentation

**FIGURE 1:**
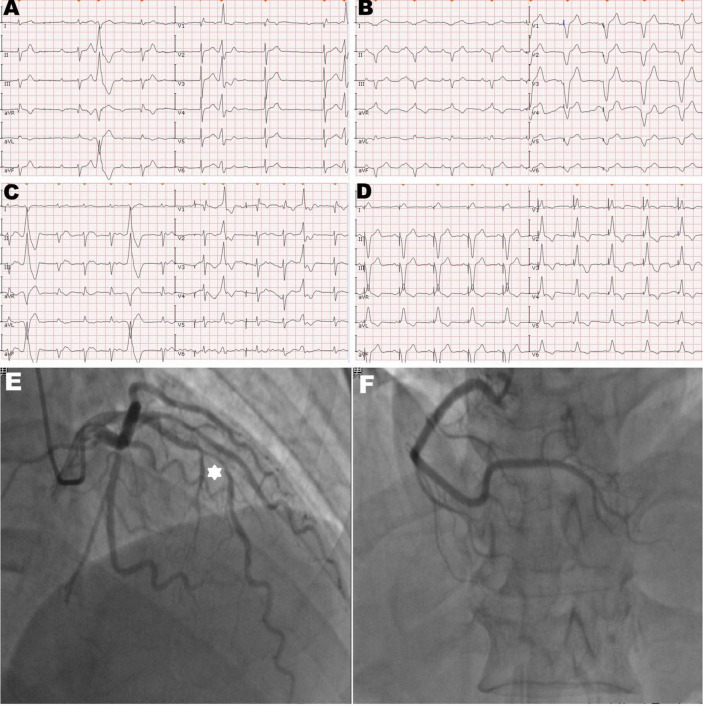
Twelve-lead electrocardiogram and coronary angiogram of the patient. **(A)** The initial electrocardiogram on the admission day showed complete atrioventricular block with frequent premature ventricular contractions. **(B)** The electrocardiogram immediately after pacemaker (PM) implantation showed the left bundle branch block pattern and the pacing model was VAT. **(C)** The electrocardiogram on the second post-operative day (POD) showed ventricular pacing failure. **(D)** The electrocardiogram after transvenous lead extraction and re-fixation showed the right bundle branch block and left axis deviation, and the right ventricular (RV) lead was refixed at the lower RV septum. **(E)** Hexagonal star symbol shows a diffuse atherosclerotic stenosis was observed in the middle segment of the left anterior descending branch, thus leading to insufficient coronary blood supply on the mid-portion of the interventricular septum. **(F)** Right coronary artery was relatively normal.

A 92-year-old man was referred to our center for syncope due to a complete atrioventricular block (Figure 1A). His medical history includes coronary atherosclerotic heart disease and chronic obstructive pulmonary disease. Coronary angiography was performed on account of effort angina 3 years ago (Figures 1E, F). Prior coronary angiography revealed a diffuse lesion (percent diameter stenosis 70–80%) in the middle segment of the left anterior descending (LAD) branch (Figure 1E). However, he refused to receive revascularization or medication treatment. During hospitalization, he had no finding of heart failure, and the transthoracic echocardiography (TTE) revealed minor mitral insufficiency and normal LV contraction (ejection fraction: 55%).

**FIGURE 2:**
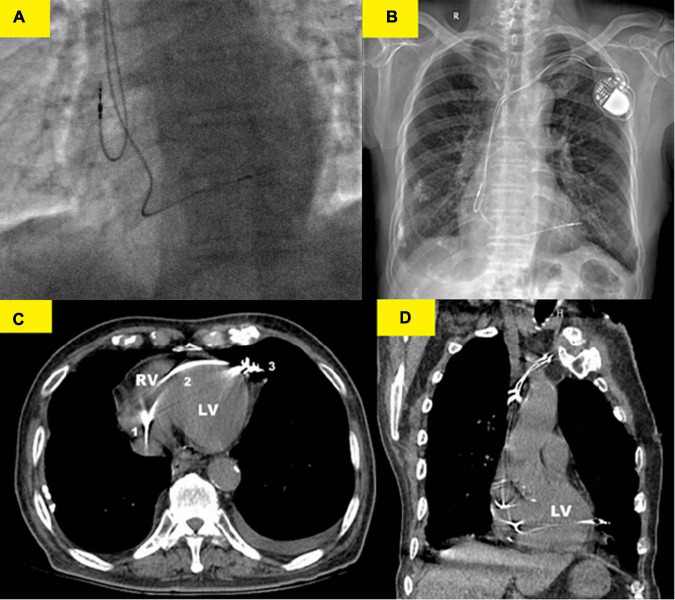
Various imaging modalities showed the lead perforation. **(A)** X-ray fluoroscopy image (right anterior oblique 30°) immediately after implantation showed that the RV lead was placed at the right ventricular (RV) apex. **(B)** Chest x-ray showed that the RV lead was beyond the left cardiac margin and clearly migrated in the left hemithorax. **(C)** Thoracic computed tomography (CT) scan showed the RV lead course from the right ventricle (1) to the left cardiac margin (3) in the patient and perforation of the interventricular septum (2) was well-evident. **(D)** Coronal CT images confirmed the RV lead migration in the left hemithorax. Thoracic CT scan should, therefore, be regarded as the gold standard for the strategical management of this complication.

We performed dual-chamber PM (HeartTone LD300D, LifeTech Scientific Corporation, Shenzhen, China) implanted *via* the left subclavian vein on the next day after admission. The implantation procedure can be summarized as follows: under local anesthesia, after a successful puncture of the left subclavian vein, the patient was implanted with a common sheath. Although a superior vena cava stenosis might exist, we did not replace the common sheath with a long sheath. Thus, the manipulation of active fixation leads was very difficult; we attempted to fix RV lead at the RV septum, but it was not as successful. Finally, two active fixation leads were, respectively, placed at the RV apex (SureScan 5076; 58 cm, Medtronic, Minneapolis, MN, USA) and the right atrial appendage (SureScan 5076; 52 cm, Medtronic, Minneapolis, MN, USA) with excellent pacing parameters (intraoperative RV lead impedance and threshold were 1,300 Ω and 1.4 V, respectively). There were no abnormalities or complications during the implantation procedure. X-ray fluoroscopy image immediately after implantation showed that the RV lead was fixed properly (Figure 2A). Electrocardiogram immediately after implantation showed the left bundle branch block pattern (Figure 1B).

However, on the second postoperative day (POD), the patient complained of unrelenting chest pain and syncope. A subsequent immediate chest X-ray identified that the RV lead tip migrated to the region outside the left cardiac margin (Figure 2B). A thoracic computed tomography (CT) scan revealed that the RV lead passed through the interventricular septum and the left LV free wall, and finally reached the left of the pericardial cavity. Furthermore, as the thoracic CT scan also showed a small amount of left pleural effusion (Figure 2B) and there were no obvious symptoms of pericardial tamponade (Figures 2C, D), we deduced that the lead had already penetrated the pericardium. Electrocardiogram and interrogation showed loss of ventricular capture (Figure 1C).

**FIGURE 3:**
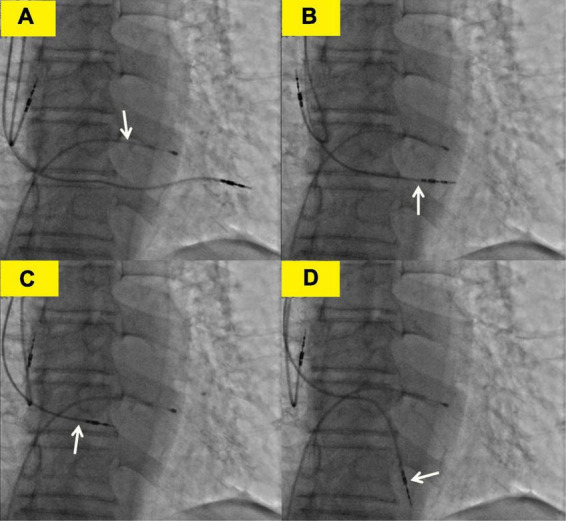
Transvenous lead extraction process. **(A)** The white arrow shows the lead of the temporary transvenous pacemaker. **(B)** The white arrow shows the right ventricular (RV) lead was screwed back to the left ventricle. **(C)** The white arrow shows the RV lead was screwed back to the right ventricle. **(D)** The white arrow shows the right ventricular lead was refixed at the lower right ventricular septum.

The subsequent patient’s management was thoroughly debated. Patients may not tolerate surgical lead extraction due to the variety of severe chronic diseases and advanced age. Therefore, on the 5th POD, we performed transvenous lead extraction under transesophageal echocardiography monitored and cardiac surgery backup. Figures 3A–D depict and describe the entire procedure in detail. Transvenous lead extraction was uncomplicated and did not have any hemodynamic instability, and intraoperative ultrasonography monitoring showed no increase in pleural effusion and pericardial effusion. The original RV lead was refixed at the lower RV septum to reduce the risk of perforation and consequent tamponade. Subsequent electrocardiogram and PM interrogation revealed the PM functioning normally with excellent pacing parameters (intraoperative RV lead impedance and threshold were 1,020 Ω and 1.0 V, respectively) (Figure 1D). On the 10th POD, the patient was discharged, and the subsequent 6 months of follow-up were uneventful.

## 3. Discussion

Left ventricular perforation caused by an RV pacing lead is a rare and potentially life-threatening complication, and the lead can migrate to the outside of the pericardium with a potential risk of vascular or pulmonary damage. Although late-onset (> 1 month) ventricular perforations have also been observed, they often started within < 24 h after device placement (4). To the best of our knowledge, no specific recommendations are known to exist for this uncommon complication. In addition, it has not yet been extensively investigated what pathophysiological mechanisms are involved (5). All of the clinical cases with this complication documented in the literature are shown in Table 1, together with their lead type, model, location, migration course, clinical characteristics, treatment, and follow-up (2, 3, 5–12).

**TABLE 1:** Summary of clinical cases of ventricular pacemaker/implantable cardioverter defibrillator (PM/ICD) leads migration in the left hemithorax including our case.

References	Sex	Age (y)	Lead type	Lead model	Lead position	Time from implantation to lead complication	Symptoms	Lead migration	Management	New lead position	Follow-up
Selcuk, et al. (6)	F	30	N/A	N/A	RV apex	2 weeks	headedness, chest pain	RV wall, pericardium, left pleura	surgical extraction	N/A	Uneventful
Migliore, et al. (7)	M	52	active fixation ICD lead	Linox SD 65/16, biotronik	RV apex	12 days	asymptomatic	septum, LV wall	surgical extraction	epicardial	Uneventful
Bohora, et al. (8)	M	44	active fixation PM lead	ICQ09B-58, vitatron	RV apex	5 days	chest pain, cough, fatigue, left hemithorax	RV wall, pericardium, left pleura	surgical extraction	epicardial	Uneventful
Kondoh, et al. (9)	M	82	active fixation PM lead	CapSureFix 5076-52, medtronic	RV sptum	3 months	chest pain	RV wall, pericardium, left pleura	surgical extraction	epicardial	Uneventful
Forleo, et al. (10)	F	81	active fixation PM lead	CapSureFix MRI, medtronic	RV apex	7 months	chest pain, left hemithorax, dyspnea, hypotension	RV wall, pericardium, left pleura	surgical extraction	RV septum	Uneventful
Pojar, et al. (11)	M	74	active fixation PM lead	N/A	RV apex	3 months	cardiogenic shock, left hemothorax, pericardial effusion	RV wall, pericardium, left pleura	surgical extraction	epicardial	Uneventful
Iribarne, et al. (2)	F	69	active fixation PM lead	CapSureFix 5076-52, medtronic	RV sptum	2 weeks	none	septum, LV wall	surgical extraction	epicardial	Uneventful
Satomi, et al. (3)	M	84	active fixation PM lead	Select secure 3830-69, medtronic	RV sptum	2 days	syncope	septum, LV wall	surgical extraction	RV septum	Uneventful
Marazzato, et al. (5)	F	78	active fixation PM lead	Solia S 60, biotronik	RV sptum	2 months	chest pain, syncope	septum, LV wall	surgical extraction	epicardial	Uneventful
Our report	M	92	active fixation PM lead	Surescan 5076-58, medtronic	RV apex	2 days	chest pain, syncope	septum, LV wall	transvenous extraction	RV septum	Uneventful

M, male; F, female; ICD, implantable cardioverter defibrillator; LV, left ventricular; N/A, not available; PM, pacemaker; RV, right ventricular.

Although the mechanisms of interventricular septal perforation are still unknown, the reported cases suggest that inadequate blood supply and the twisting motion of the septal musculature from beat to beat may be associated with this complication (5). Besides, active fixation leads positioned on the thin-walled RV apex also seem to be related to an increased risk of left-sided lead migration, according to earlier observations on patients with lead perforation. Indeed, in this case, the perforation portion of the septal was the coronary angiogram that had previously shown insufficient coronary blood supply. Although active fixation leads positioned in the RV apex result in greater stability, the straight shape of the distal RV lead may bring about significant pressure perpendicularly transferred to the ventricular wall (13). Moreover, features of the lead (SureScan 5076; 58 cm, Medtronic) also seem to play a pivotal role in our case. This lead’s non-retractable bare screw and substantial tipping load may have created excessive stress on the ventricular wall (12). Finally, excessive loop of the RV lead may generate more tension in the ventricular wall, which is also a potential cause of ventricular perforation. In this regard, to prevent the recurrence of this lead implantation complication, a leadless PM should be used in conjunction with a functioning AAI system as an alternate treatment.

A previous study found that pericardial effusion and tamponade only occurred in 38 and 19% of patients (14). However, detecting cardiac perforation brought on by ventricular leads with clinical symptoms was not difficult. Therefore, in instances with unclear results and/or a lack of symptoms, thoracic CT scans are essential to determine if the pleural or LV free wall is implicated and to establish an appropriate diagnosis.

According to a consensus endorsed by the American Heart Association, transvenous lead extraction is not the preferred strategy for patients with cardiac perforation due to pacing/defibrillation lead (15). As indicated in Table 1, surgical lead extraction was carried out without incident in all instances of lead migration in the left hemithorax. Still, for patients who may not tolerate surgical extraction or refuse surgical extraction, transvenous lead extraction could be a potential alternative, especially in recent implantations. It is a less invasive method than open surgery that avoids the potential complications of sternotomy as well as long-term hospital stays (16).

In this report, to the best of our knowledge, we first demonstrated the transvenous lead extraction to treat the lead migration in the left hemithorax. From our experience, spontaneous closure of the perforated site with myocardial contraction is likely to occur immediately after lead withdrawal in LV or RV perforation. Therefore, transvenous lead extraction is feasible, but considering its high-risk characteristics, the whole procedure should be performed by an experienced intervention cardiologist specializing in transvenous lead extraction, preferably in a hybrid operating room, under careful hemodynamic and transesophageal echocardiographic monitoring with a cardiac surgical backup. In addition, transvenous lead extraction should be followed by a new lead implantation or re-fixing of the perforating lead in a different location.

## 4. Conclusion

The presence of unrelenting chest pain should consider the possibility of a lead migration in the left hemithorax in cases of suspected cardiac perforation. To assess whether the pleural or LV free wall is involved, thoracic CT scans are essential. With respect to lead extraction, transvenous lead extraction with cardiac surgery backup can be feasible. In addition, less traumatic passive-fixation leads or leadless PM may be employed in these high-risk individuals with evidence of non-revascularizable coronary atherosclerotic heart disease and unequivocal need for PM/ICD implantation.

## Data availability statement

The original contributions presented in this study are included in the article/Supplementary material, further inquiries can be directed to the corresponding author.

## Ethics statement

Written informed consent was obtained from the individual(s) for the publication of any potentially identifiable images or data included in this article. The patient consent form was read and signed by the patient.

## Author contributions

XH: methodology, investigation, formal analysis, and writing—original draft. HZ: conceptualization and visualization. X-ML: investigation and writing—review. X-LL: project administration and supervision. All authors contributed to the article and approved the submitted version.

## Supplementary material

Supplementary Figure 1 Transvenous lead extraction process.
