# Supplementary figures and images for: Case report: Left ventricular perforation caused by right ventricular pacemaker lead
Source: Front Cardiovasc Med. 2023 Jan 12;9:1089694. doi: 10.3389/fcvm.2022.1089694 (PMC9877449; doi:10.3389/fcvm.2022.1089694)

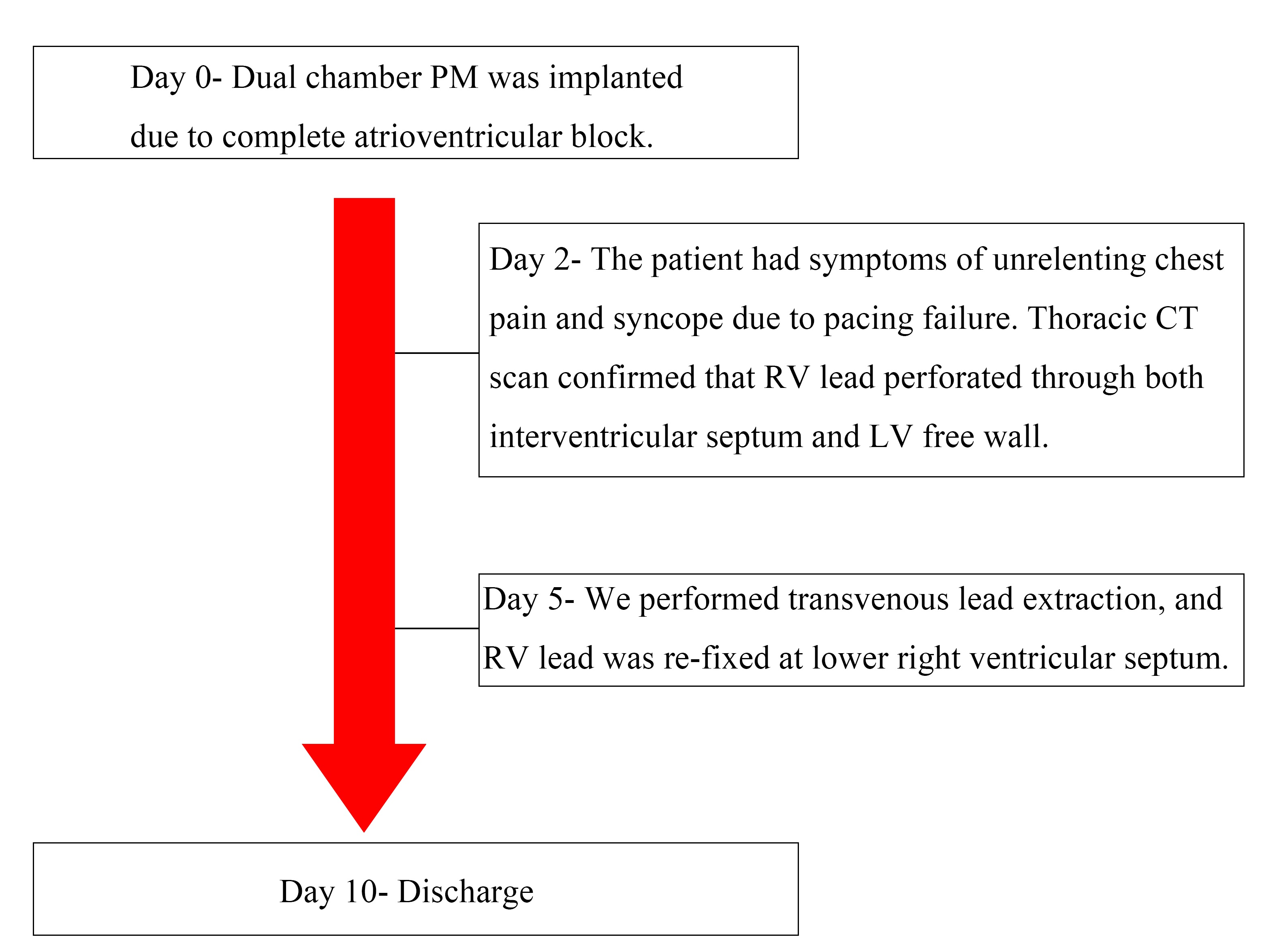

Supplement: Supplementary Figure 1 — Transvenous lead extraction process. [file Image_1.jpeg]
